# Supplementary material for: Visible implant elastomer (VIE) success in early larval stages of a tropical amphibian species
Source: PeerJ. 2020 Aug 10;8:e9630. doi: 10.7717/peerj.9630 (PMC7425637; doi:10.7717/peerj.9630)
Supplement: Supplemental Information 5 — (Table 1) Mathematical formulation for each of the models considered in JAGS. (Table 2-3) Posterior distributions of Bayesian analysis. (Table 4) Uninformative priors used for every parameter & model. (Table 5) Chain convergence estimates. (Figure 1-2) Posterior distribution and chain convergence of models. (Figure 3) Weekly growth rate across development. (Figure 4) Tag presence across development. (Figure 5) Tagged recently hatched tadpoles, prior to transport. [file peerj-08-9630-s005.docx]

Updated Supplementary Materials VIE paper

Table 1. Model descriptions

| Model Name | Description |
| --- | --- |
| M1 | Constant probabilities of VIE tag retention and observation |
| M2 | Week of development dependent probabilities of VIE tag retention and observation |
| M3 | Probabilities of VIE tag retention and observation dependent on the week of development and, through a random effect, individual identity. |
| M4 | Probabilities of VIE tag retention and observation dependent on the week of development and weight at the moment of tagging. |
| M5 | Probabilities of VIE tag retention and observation dependent on the week of development, weight at the moment of tagging and, through a random effect, individual identity. |

Table 2. DIC score comparisons between models

| Model Name | DIC score | ∆DIC with respect to the best model |
| --- | --- | --- |
| M1 | 154.44 | 0 |
| M2 | 165.98 | 11.53 |
| M3 | 202.47 | 48.03 |
| M4 | 179.86 | 25.42 |
| M5 | 187.62 | 33.18 |

Table 1. Mathematical formulation for each of the models considered in JAGS.

| Model | Mathematical formulation |
| --- | --- |
| M1 | $Retention \sim B\left( \phi\right)$ |
|  | $Observation \sim B(p)$ |
| M2 | $\phi_{t}=\frac{1}{1+e^{-(\alpha_{\phi}+\beta_{\phi}t)}}$  ${Retention}_{t} \sim B\left( \phi_{t} \right)$ |
|  | $p_{t}=\frac{1}{1+e^{-(\alpha_{p}+\beta_{p}t)}}$  ${Observation}_{t} \sim B\left( p_{t} \right)$ |
| M3 | $\alpha_{\phi_{id}}\sim N(0,\sigma_{\alpha_{\phi_{id}}}^{2})$  $\beta_{\phi_{id}}\sim N(0,\sigma_{\beta_{\phi_{id}}}^{2})$  $\phi_{t,id}=\frac{1}{1+e^{-(\alpha_{\phi}+\alpha_{\phi_{id}}+\left( \beta_{\phi}+\beta_{\phi_{id}} \right) t)}}$  ${Retention}_{t,id} \sim B\left( \phi_{t,id} \right)$ |
|  | $\alpha_{p_{id}}\sim N(0,\sigma_{\alpha_{p_{id}}}^{2})$  $\beta_{p_{id}}\sim N(0,\sigma_{\beta_{p_{id}}}^{2})$  $p_{t,id}=\frac{1}{1+e^{-\left( \alpha_{p}+\alpha_{p_{id}}+\left( \beta_{p}+\beta_{p_{id}} \right) t \right)}}$  ${Observation}_{t,id} \sim B\left( p_{t,id} \right)$ |
| M4 | $\phi_{t,w}=\frac{1}{1+e^{-(\alpha_{\phi}+\beta_{\phi_{t}}t+ \beta_{\phi_{w}}w)}}$  ${Retention}_{t,w} \sim B\left( \phi_{t,w} \right)$ |
|  | $p_{t,w}=\frac{1}{1+e^{-(\alpha_{\phi}+\beta_{\phi_{t}}t+ \beta_{\phi_{w}}w)}}$  ${Retention}_{t,w} \sim B\left( p_{t,w} \right)$ |
| M5 | $\alpha_{\phi_{id}}\sim N(0,\sigma_{\alpha_{\phi_{id}}}^{2})$  $\beta_{\phi_{id}}\sim N(0,\sigma_{\beta_{\phi_{id}}}^{2})$  $\phi_{t,id,w}=\frac{1}{1+e^{-(\alpha_{\phi}+\alpha_{\phi_{id}}+\left( \beta_{\phi_{t}}+\beta_{\phi_{id}} \right) t+\beta_{\phi_{w}}w)}}$  ${Retention}_{t,id,w} \sim B\left( \phi_{t,id,w} \right)$ |
|  | $\alpha_{p_{id}}\sim N(0,\sigma_{\alpha_{p_{id}}}^{2})$  $\beta_{p_{id}}\sim N(0,\sigma_{\beta_{p_{id}}}^{2})$  $p_{t,id,w}=\frac{1}{1+e^{-\left( \alpha_{p}+\alpha_{p_{id}}+\left( \beta_{p_{t}}+\beta_{p_{id}} \right) t+\beta_{p_{w}}w \right)}}$  ${Observation}_{t,id,w} \sim B\left( p_{t,id,w} \right)$ |

Table 2. Posterior distribution results of the M1 model

| Parameter | Mean | SD | 2.5% Q. | 97.5% Q. |
| --- | --- | --- | --- | --- |
| $\phi$ | 0.9572 | 0.01301 | 0.9293 | 0.9800 |
| $p$ | 0.9274 | 0.01775 | 0.8904 | 0.9581 |
| Deviance | 118.3336 | 8.56818 | 105.6864 | 137.3522 |

Table 3. Posterior distribution results of the M2 model

| Parameter | Mean | SD | 2.5% Q. | 97.5% Q. | Significance |
| --- | --- | --- | --- | --- | --- |
| $\alpha_{\phi}$ | 3.08207 | 0.51868 | 2.127 | 4.1696 | * |
| $\beta_{\phi}$ | 3.99445 | 0.72535 | 2.7159 | 5.56701 | * |
| $\alpha_{p}$ | -0.09068 | 0.08951 | -0.2685 | 0.08852 |  |
| $\beta_{p}$ | -0.12104 | 0.11338 | -0.3400 | 0.10763 |  |
| Deviance | 120.53643 | 9.63689 | 105.7178 | 142.22163 |  |

Table 4. Uninformative priors used for every parameter & model. * Indicates that only positive values were sampled from the normal distribution for the random effects model. The standard deviation of the normal distributions from which the random effect of individual identity were sampled can only assume positive values.

| Model | Parameter | Prior distribution |
| --- | --- | --- |
| M1 | $\phi$ | Uniform distribution with 0 and 1 as limits |
|  | $p$ | Uniform distribution with 0 and 1 as limits |
| M2 | $\alpha_{\phi}$ | Normal distribution, mean = 0, sd = 10^-4^ |
|  | $\beta_{\phi}$ | Normal distribution, mean = 0, sd = 10^-4^ |
|  | $\alpha_{p}$ | Normal distribution, mean = 0, sd = 10^-4^ |
|  | $\beta_{p}$ | Normal distribution, mean = 0, sd = 10^-4^ |
| M3 | $\alpha_{\phi}$ | Normal distribution, mean = 0, sd = 10^-4^ |
|  | $\beta_{\phi}$ | Normal distribution, mean = 0, sd = 10^-4^ |
|  | $\alpha_{p}$ | Normal distribution, mean = 0, sd = 10^-4^ |
|  | $\beta_{p}$ | Normal distribution, mean = 0, sd = 10^-4^ |
|  | $\sigma_{\alpha_{\phi}}$ | Normal distribution*, mean = 0, sd = 10^-4^ |
|  | $\sigma_{\beta_{\phi}}$ | Normal distribution*, mean = 0, sd = 10^-4^ |
|  | $\sigma_{\alpha_{p}}$ | Normal distribution*, mean = 0, sd = 10^-4^ |
|  | $\sigma_{\beta_{p}}$ | Normal distribution*, mean = 0, sd = 10^-4^ |
| M4 | $\alpha_{\phi}$ | Normal distribution, mean = 0, sd = 10^-4^ |
|  | $\beta_{\phi_{t}}$ | Normal distribution, mean = 0, sd = 10^-4^ |
|  | $\beta_{\phi_{w}}$ | Normal distribution, mean = 0, sd = 10^-4^ |
|  | $\alpha_{p}$ | Normal distribution, mean = 0, sd = 10^-4^ |
|  | $\beta_{p_{t}}$ | Normal distribution, mean = 0, sd = 10^-4^ |
|  | $\beta_{p_{w}}$ | Normal distribution, mean = 0, sd = 10^-4^ |
| M3 | $\alpha_{\phi}$ | Normal distribution, mean = 0, sd = 10^-4^ |
|  | $\beta_{\phi_{t}}$ | Normal distribution, mean = 0, sd = 10^-4^ |
|  | $\beta_{\phi_{w}}$ | Normal distribution, mean = 0, sd = 10^-4^ |
|  | $\alpha_{p}$ | Normal distribution, mean = 0, sd = 10^-4^ |
|  | $\beta_{p_{t}}$ | Normal distribution, mean = 0, sd = 10^-4^ |
|  | $\beta_{p_{w}}$ | Normal distribution, mean = 0, sd = 10^-4^ |
|  | $\sigma_{\alpha_{\phi}}$ | Normal distribution*, mean = 0, sd = 10^-4^ |
|  | $\sigma_{\beta_{\phi}}$ | Normal distribution*, mean = 0, sd = 10^-4^ |
|  | $\sigma_{\alpha_{p}}$ | Normal distribution*, mean = 0, sd = 10^-4^ |
|  | $\sigma_{\beta_{p}}$ | Normal distribution*, mean = 0, sd = 10^-4^ |

Table 5. Chain convergence estimated through the Potential scale reduction factor (PSRF) and effective sample size of the parameters from models M1 and M2.

| Model | Parameter | PSRF (Point est.) | PSRF (Upper C.I.) | Effective Size |
| --- | --- | --- | --- | --- |
| M1 | $\phi$ | 1 | 1.00 | 2054.260 |
|  | $p$ | 1 | 1.00 | 1802.229 |
| M2 | $\alpha_{\phi}$ | 1 | 1.00 | 1276.573 |
|  | $\beta_{\phi}$ | 1 | 1.00 | 1145.789 |
|  | $\alpha_{p}$ | 1 | 1.01 | 1311.156 |
|  | $\beta_{p}$ | 1 | 1.01 | 1240.023 |

**A**
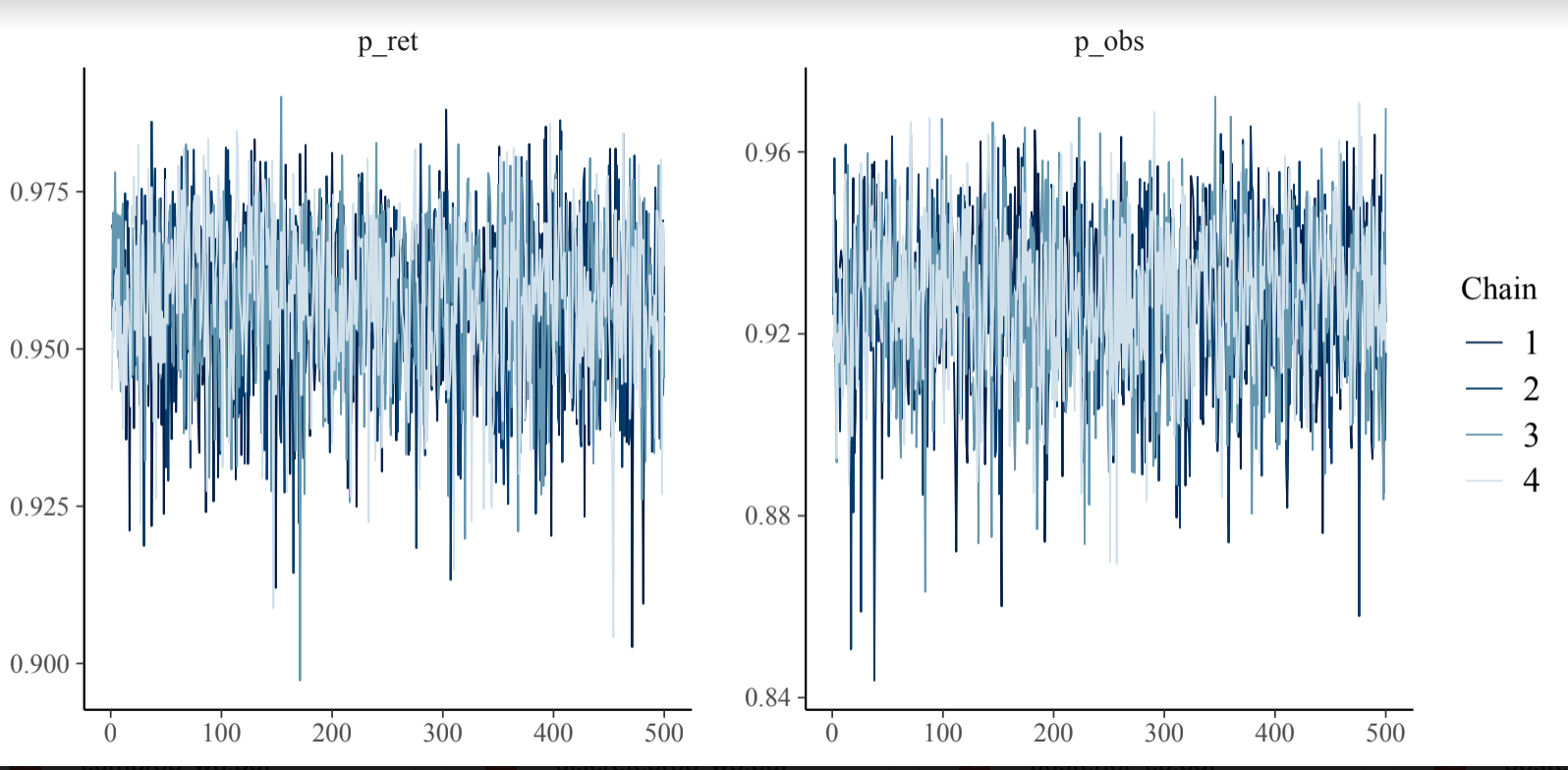
. **B**.
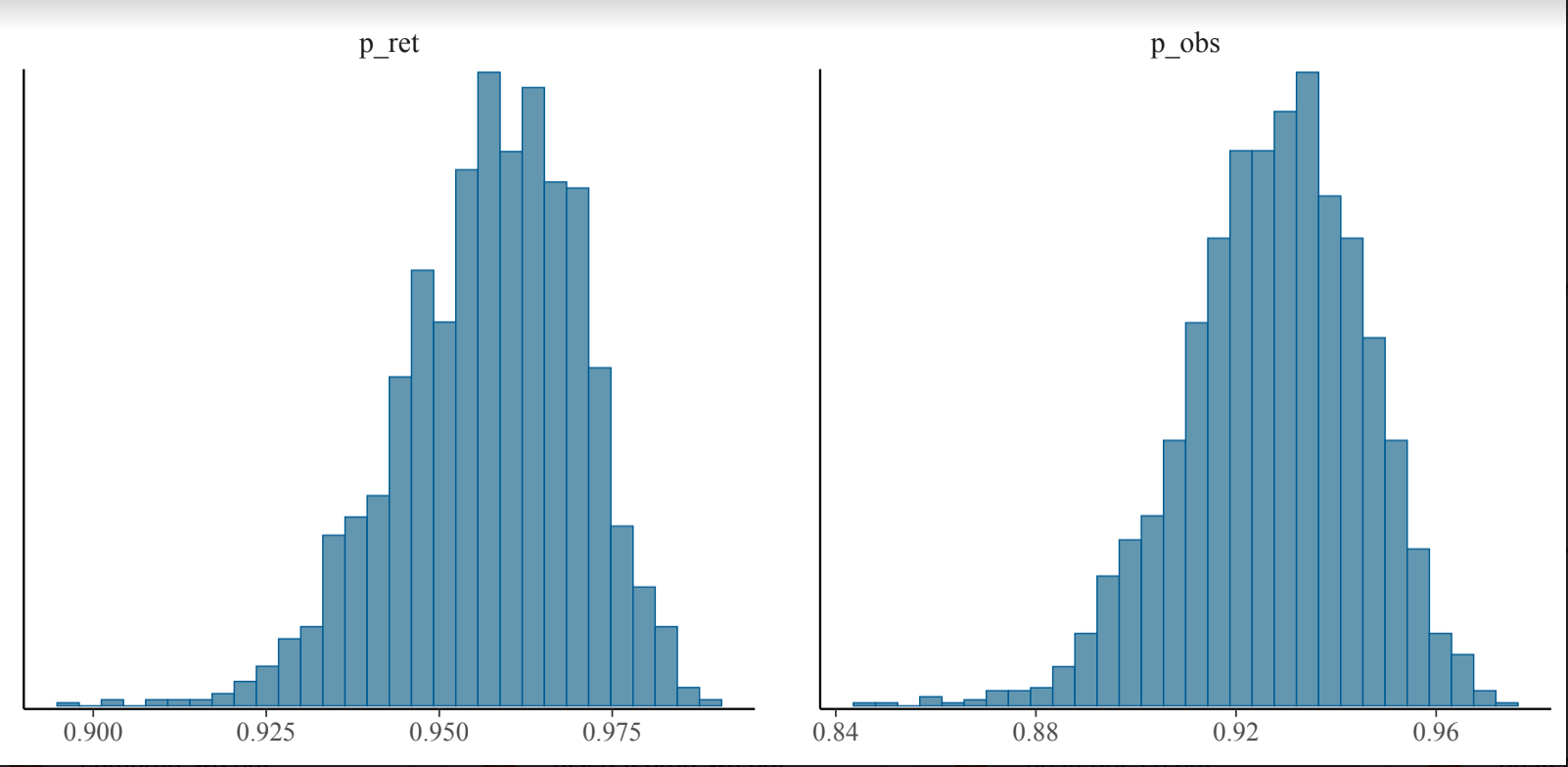


Supplementary figure 1. Posterior distribution and chain convergence of M1. Model assumed constant probabilities of observation and retention. Panel A is a trace plot indicating the convergence of 4 chains based on MCMC sampling and panel B is the posterior distribution generated by model function; *ret* and *obs* denote retention and observation, respectively.

**A**.
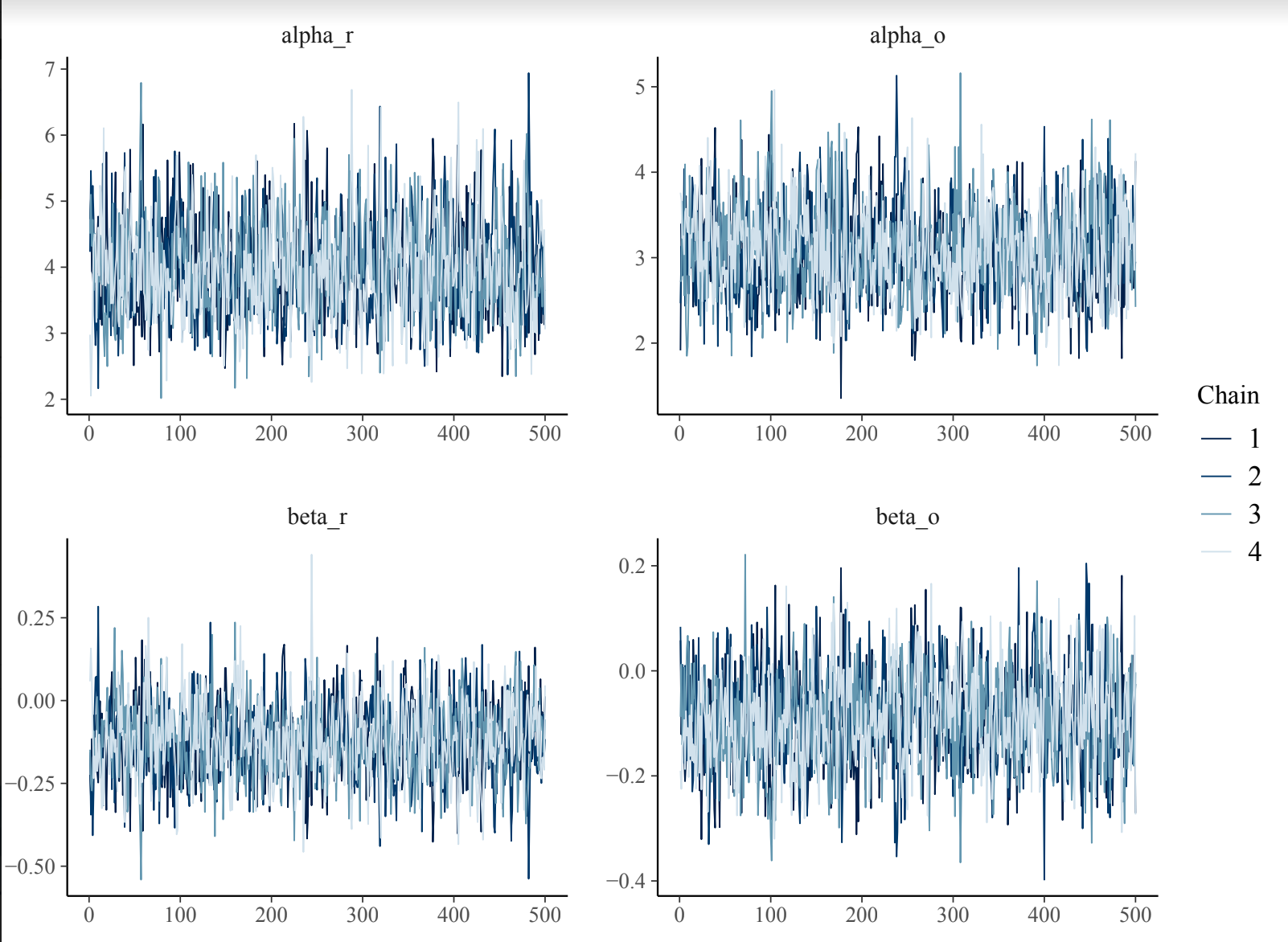
**B**.
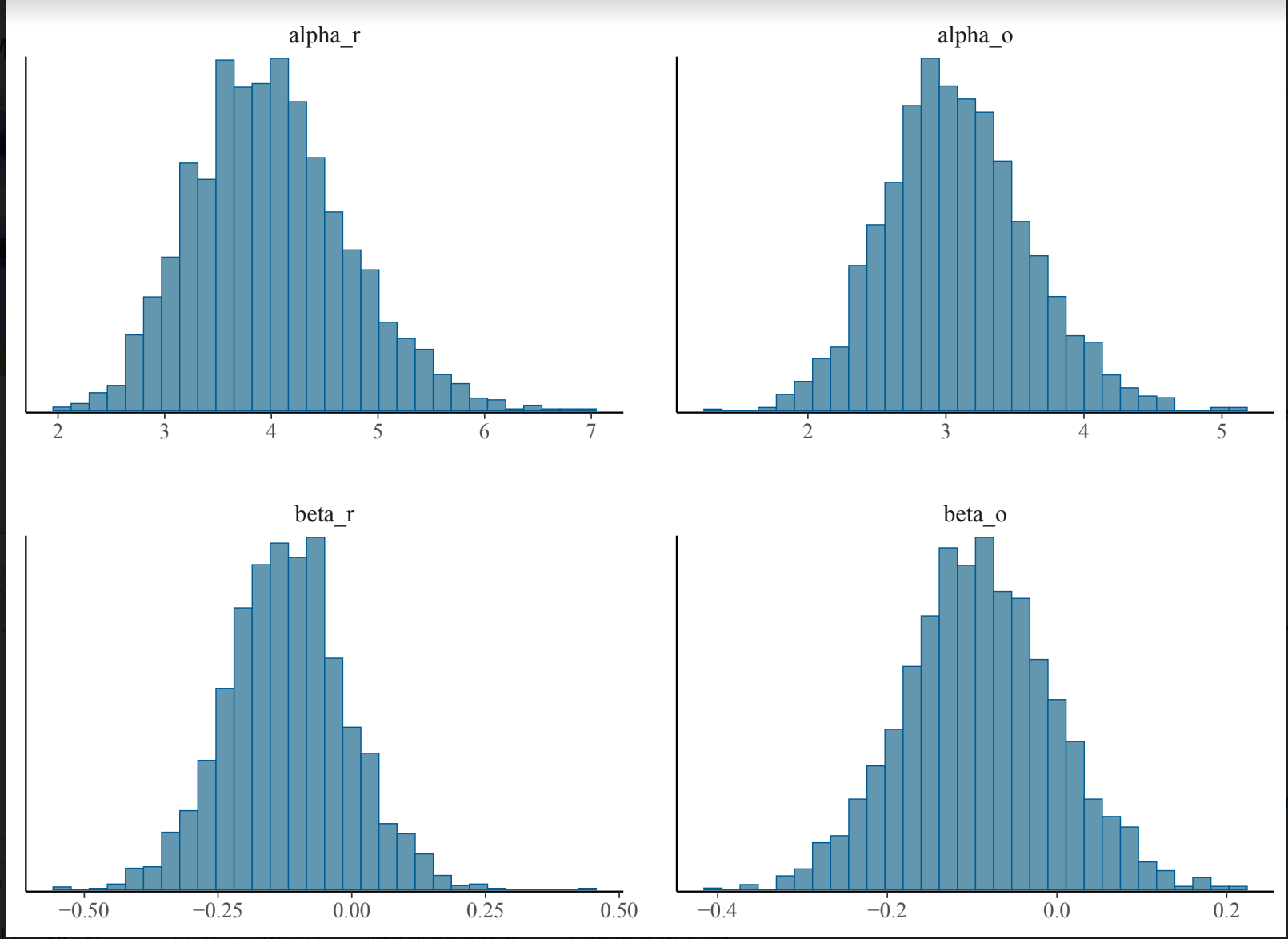


Supplementary figure 2. Posterior distribution and chain convergence of M2. Model considered a week effect on both probabilities using a logit link function. Panel A is a trace plot indicating the convergence of 4 chains based on MCMC sampling and panel B visualizes the posterior distribution generated by model function; *r* and *o* denote retention and observation, respectively.

| **Fixed Effects** | Estimate | Std. Error | t-value |
| --- | --- | --- | --- |
| **Intercept** | 26.3323 | 3.1739 | 8.296 |
| **TreatmentTag** | -3.2484 | 3.0587 | -1.062 |
| **Week** | -1.5019 | 0.1983 | -7.575 |

Table 6A. Summary output of LMM of growth rate model.

| **Fixed Effects** | Sum Sq | Mean Sq | DenDF | F values | Pr (>F) | Significance |
| --- | --- | --- | --- | --- | --- | --- |
| Treatment | 533.4 | 533.4 | 37.97 | 1.1212 | 0.2963 |  |
| Week | 26854.6 | 26854.6 | 415.88 | 56.4498 | 3.563e-13 | *** |

Table 6B. Type III ANOVA Table with Kenward-Roger's method for LMM growth rate model.

| **Fixed Effects** | Coef | se(coef) | z | Pr (>\| z \|) |
| --- | --- | --- | --- | --- |
| **TreatmentTag** | -0.1181 | 0.3961 | -0.298 | 0.766 |

Table 7A. Summary output of Cox Proportional Hazards Model

| **Fixed Effects** | loglik | Chisq | Df | Pr (>\| Chi \|) |
| --- | --- | --- | --- | --- |
| Null | -129.91 |  |  | 1.1212 |
| Treatment | -129.91 | 0.0879 | 1 | 56.4498 |

Table 7B. ANOVA Table for Cox Model


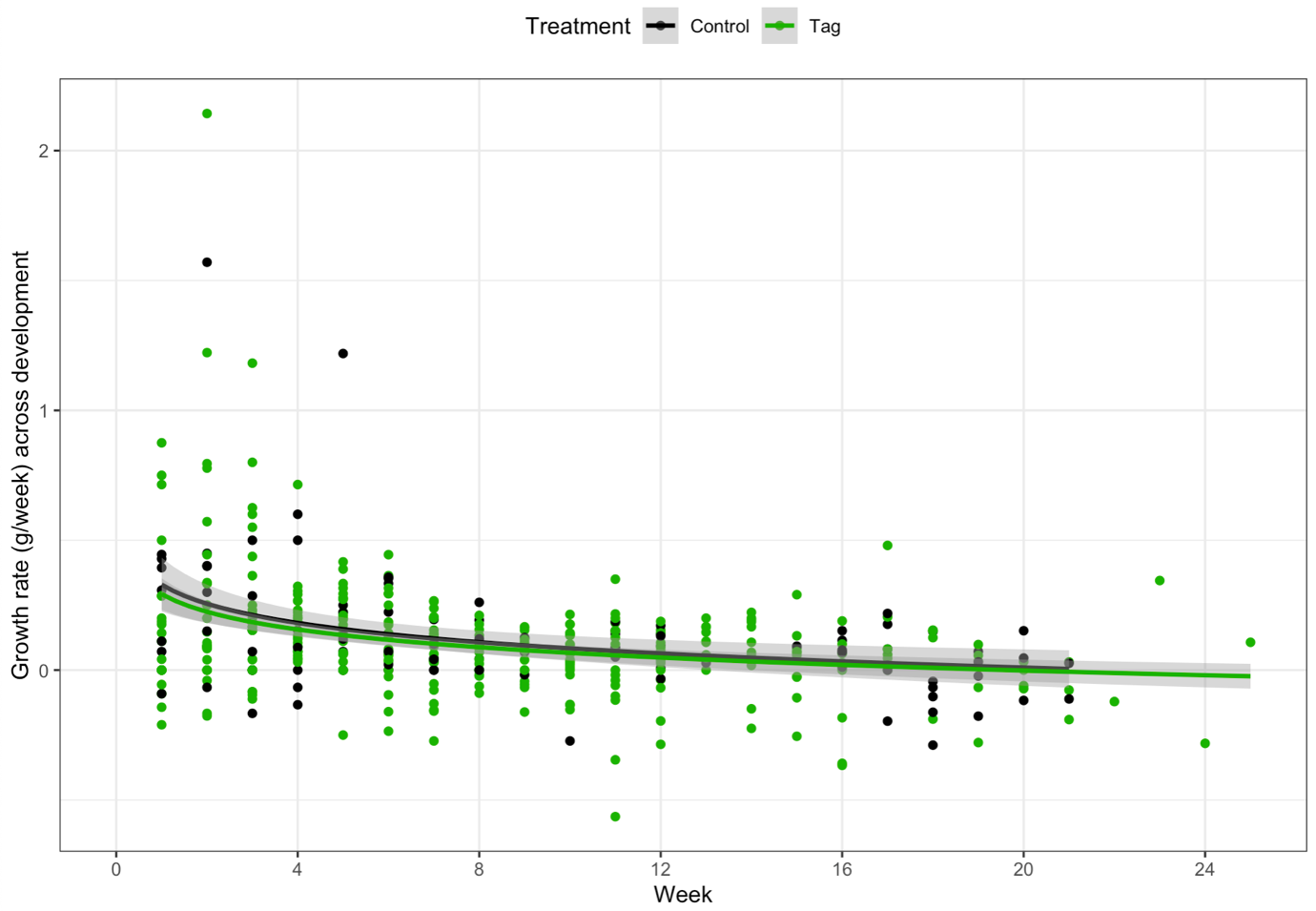


Supplementary figure 3. Weekly growth rate across development. Weekly growth rate decreases significantly over time. Grey bands represent 95% confidence intervals drawn by GLM smoother with a y ~log(x) formula.


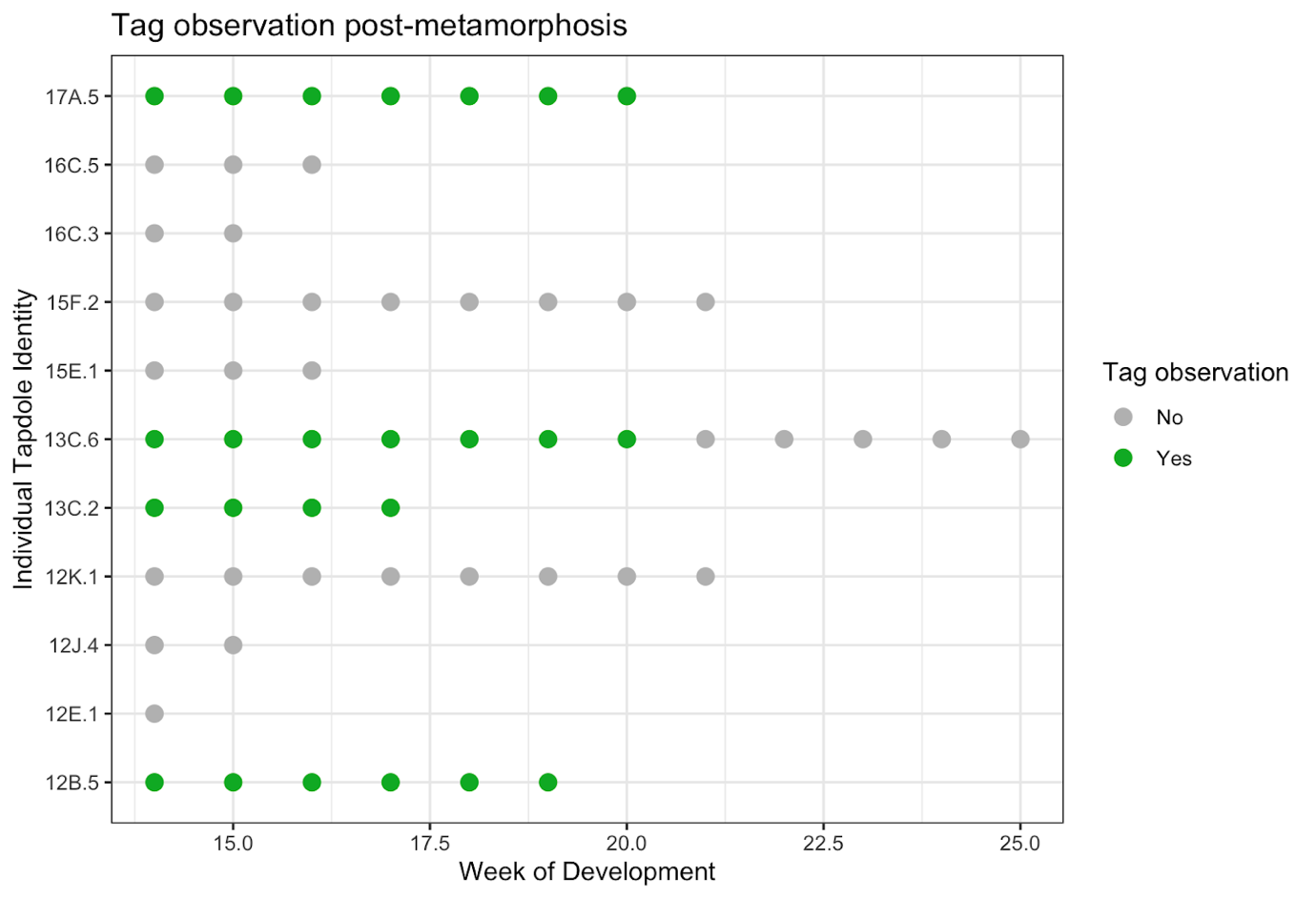


Supplementary figure 4. Post-metamorphic tag success. From the 11 tadpoles who made it past metamorphosis (> 13 weeks), only four retained their tag (36%).


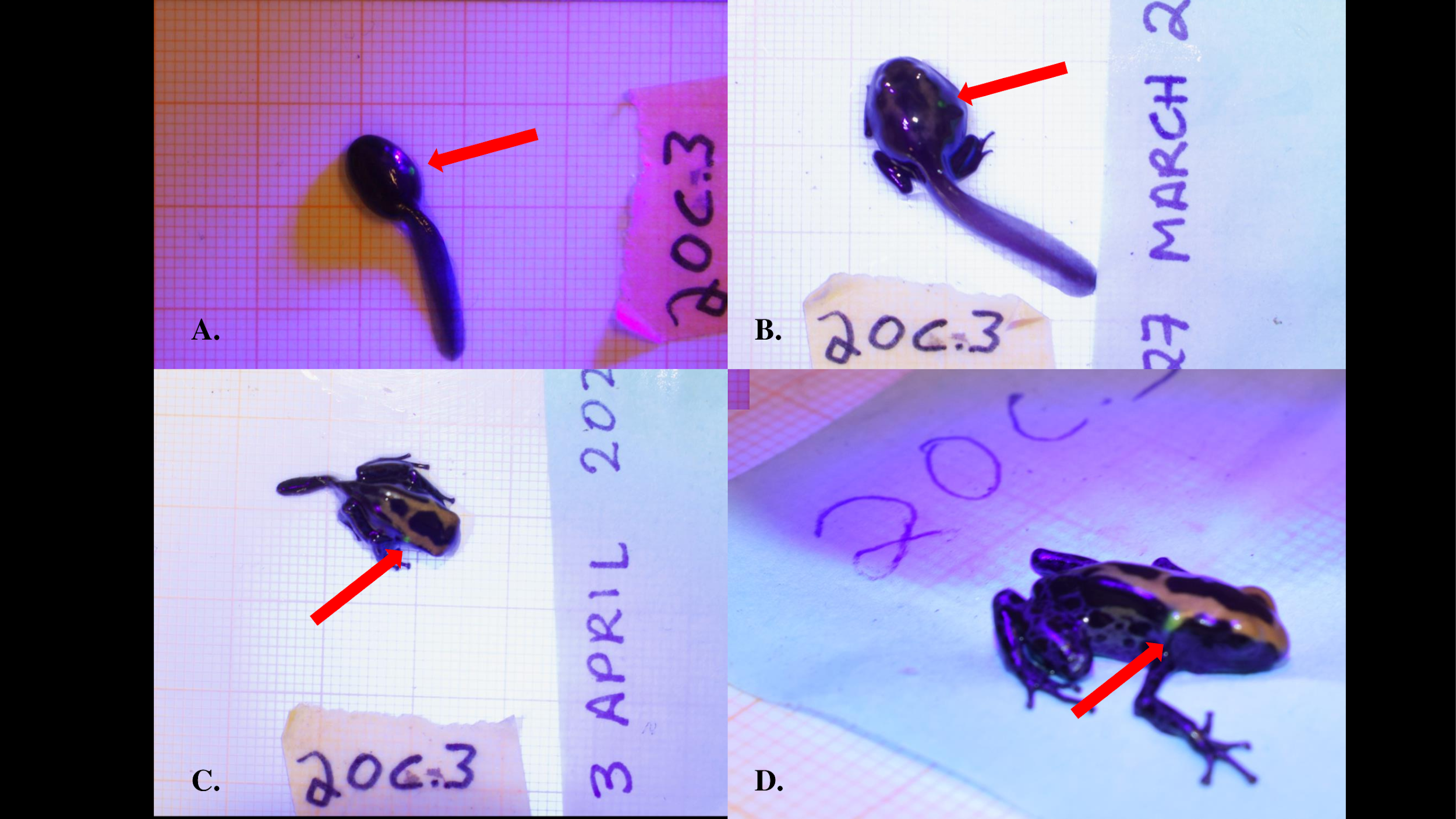


Supplementary figure 5. Fluorescent green VI Elastomer tag inserted dorsally on *Dendrobates tinctorius* shown on the same individual as (a) an early stage larvae, (b) a metamorph, (c) a late stage metamorph, and (d) a recently metamorphosed juvenile. All photos taken with Nikon DS5300 DSLR on 1 x 1 mm background under UV light to enhance tag detection.


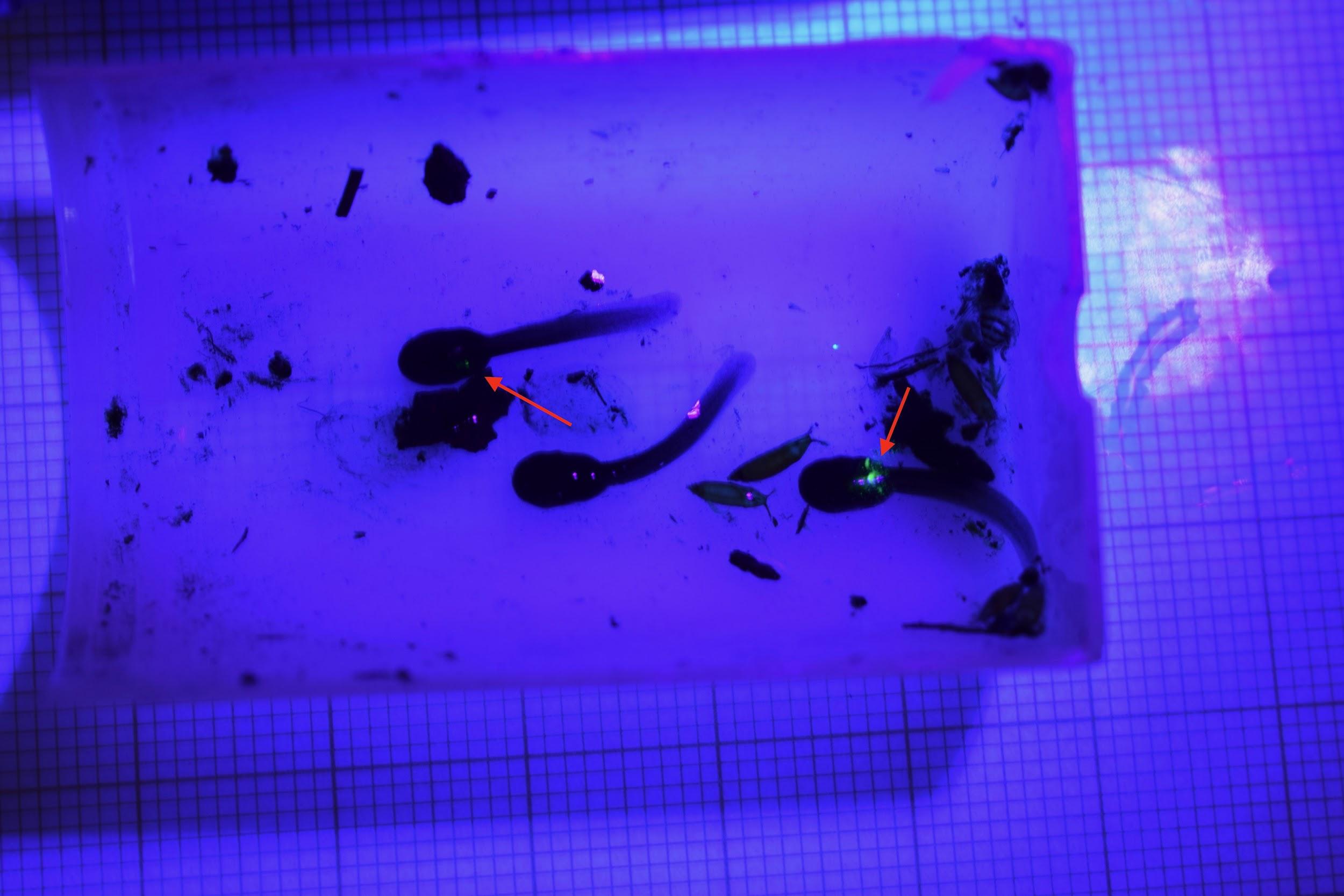


Supplementary figure 6. Recently hatched tadpoles that were successfully transported by father. Red arrows indicate tagged individuals. Photograph is taken after the initial tagging session, but before the transport event.
